# NAPE-PLD deletion in stress-TRAPed neurons results in an anxiogenic phenotype

**DOI:** 10.1038/s41398-023-02448-9

**Published:** 2023-05-06

**Authors:** Margaryta Tevosian, Hristo Todorov, Ermelinda Lomazzo, Laura Bindila, Natsuo Ueda, Davide Bassetti, Davide Warm, Sergei Kirischuk, Heiko J. Luhmann, Susanne Gerber, Beat Lutz

**Affiliations:** 1 Institute of Physiological Chemistry, University Medical Center of the Johannes Gutenberg University, Mainz, Germany; 2 Leibniz Institute for Resilience Research (LIR), Mainz, Germany; 3 Institute of Human Genetics, University Medical Center of the Johannes Gutenberg University, Mainz, Germany; 4 Department of Biochemistry, Kagawa University School of Medicine, Kagawa, Japan; 5 Department of Mathematics, Technical University of Kaiserslautern, Kaiserslautern, Germany; 6 Institute of Physiology, University Medical Center of the Johannes Gutenberg University, Mainz, Germany

**Keywords:** Molecular neuroscience, Depression

## Abstract

Anandamide (AEA) is an endogenous ligand of the cannabinoid CB1 and CB2 receptors, being a component of the endocannabinoid signaling system, which supports the maintenance or regaining of neural homeostasis upon internal and external challenges. AEA is thought to play a protective role against the development of pathological states after prolonged stress exposure, including depression and generalized anxiety disorder. Here, we used the chronic social defeat (CSD) stress as an ethologically valid model of chronic stress in male mice. We characterized a genetically modified mouse line where AEA signaling was reduced by deletion of the gene encoding the AEA synthesizing enzyme N-acyl-phosphatidylethanolamine-hydrolyzing phospholipase D (NAPE-PLD) specifically in neurons activated at the time of CSD stress. One week after the stress, the phenotype was assessed in behavioral tests and by molecular analyses. We found that NAPE-PLD deficiency in neurons activated during the last three days of CSD stress led to an increased anxiety-like behavior. Investigating the molecular mechanisms underlying this phenotype may suggest three main altered pathways to be affected: (i) desensitization of the negative feedback loop of the hypothalamic-pituitary-adrenal axis, (ii) disinhibition of the amygdala by the prefrontal cortex, and (iii) altered neuroplasticity in the hippocampus and prefrontal cortex.

## Introduction

The endocannabinoid (eCB) system consists of the cannabinoid type 1 and type 2 receptors (CB1, CB2), their main endogenous ligands 2-arachidonoyl glycerol (2-AG) and N-arachidonoyl ethanolamine (anandamide, AEA), and the synthesizing and degrading enzymes of eCBs. At synapses, the eCB system was originally proposed to be mainly involved in retrograde suppression of neurotransmitter release, mediated by postsynaptically synthesized and released 2-AG and binding to the G_i/o_ protein-coupled CB1 at the presynaptic site. Recent insights, though, have revealed a much higher complexity of how eCBs influence synaptic transmission and plasticity, including AEA, also due to the involvement of glial cells, such as astrocytes [1, 2]. AEA belongs to the family of N-acylethanolamines (NAEs), which are synthesized by the Ca^2+^ dependent enzyme N-acyl phosphatidylethanolamine phospholipase D (NAPE-PLD), which is dominantly expressed at the presynapse [3]. AEA can be produced on demand, whereby synthesized overshoots are rapidly eradicated by degrading enzymes, such as fatty acid amide hydrolase (FAAH) [4].

NAPE-PLD deficient mice were independently generated by three groups [5–7]. While AEA levels were not reduced in the NAPE-PLD mutant line generated by the Cravatt’s group [6], AEA was significantly reduced in the lines created by Ueda’s group [7] and Luquet’s group [5]. In our study, we used the NAPE-PLD^fl/fl^ mouse line by Ueda’s group [7]. Some studies on NAPE-PLD deficient mice from Cravatt’s laboratory showed that AEA levels in the brain of these mice were not different from wild-type littermates [8], suggesting alternative synthesis pathways involving other enzymes: the glycerophosphodiesterase (GDE) enzyme family, GDE1, GDE4, and GDE7, as well as α/β-hydrolase domain-containing protein 4 (ABHD4) [9]. Furthermore, NAPE-PLD deficiency was shown to lead to lipid changes beyond AEA, including different NAEs [10].

Stress can be defined as an external or internal challenge that is perceived as threatful and evokes specific physiological and behavioral responses in an organism [11]. Prolonged exposure to an aversive environment causes chronic stress, which can exhaust the resources, leading to the development of pathological states, such as depression and generalized anxiety disorders. Several studies involving the CB1 antagonist rimonabant and CB1 deficient mice introduced the notion that the eCB system exerts an inhibitory action on the hypothalamic-pituitary-adrenal (HPA) axis [12–15]. eCBs reportedly act at the site of fast, non-genomic negative feedback [15]. CB1 is expressed in many brain regions involved in the processing of anxiety and stress, including the hippocampus (Hip), the prefrontal cortex (PFC), the bed nucleus of the stria terminalis (BNST), the basolateral (BLA) and central amygdala (CeA) and various hypothalamic nuclei (Hypo) [16]. BNST integrates neuronal inputs from these regions for the inhibitory control of emotional stress induced by HPA axis activity [17]. eCB signaling in the BLA modulates both excitatory and inhibitory neurotransmission, and the content of 2-AG and AEA in the BLA is modulated in response to stress [18].

Studies on stress-induced human psychopathologies further pinpoint an important role of AEA in the stress response. For example, the human C385A single-nucleotide polymorphism (SNP) in the *Faah* gene was studied with regards to pain insensitivity and elevated AEA levels [19], as well as in more moderate response to stress in posttraumatic stress disorder (PTSD) patients [20–23].

AEA content in the amygdala, medial PFC (mPFC), Hip, and Hypo was shown to decrease upon chronic stress exposure [24]. These changes are linked to the concomitant elevation of FAAH activity in these regions, resulting in enhanced hydrolysis of AEA [25]. It is hypothesized that reduction of AEA in BLA results in inadequate gating of excitatory inputs leading to increased glutamate release, causing imbalanced behavioral response to stress. Whole-brain AEA levels were shown to negatively correlate with anxiety-like behavior [26]. Interestingly, pharmacological inhibition or genetic ablation of FAAH leads to the absence of anxiety-like behavior in mice following stress [27], whereas the anxiolytic effect is only observed during aversive testing conditions or stress exposure and absent at baseline non-stressful conditions [28].

The mPFC, a region responsible for top-down control of the emotional response, is highly intertwined with AEA content and responses to stress [29]. As mentioned above, chronic stress results in a decrease of AEA content in the mPFC, leading to disinhibition and basal hyperactivity of the HPA axis. Moreover, low levels of AEA correlate with hypersecretion of corticosterone (CORT). This effect is reversed by elevation of AEA through FAAH inhibition [24]. It has also been observed that cannabinoid CB1 receptor (*Cnr1*) mRNA is upregulated in the mPFC after chronic stress, possibly to compensate for the deficiency of AEA [30]. The maintenance of the AEA levels in the mPFC could buffer exaggerated responses to stress via mPFC top-down control and thus could be an important factor for stress resilience [31, 32].

Although the involvement of AEA in fear conditioning and extinction paradigms [33–35], as well as its role in the response to chronic restraint stress and chronic unpredictable stress (CUS) [36–38] has been well studied, the involvement of AEA in the framework of chronic social defeat and resulting behavior alterations has not yet been investigated.

The widely used CSD model combines elements of both physiological and psychological stress, follows construct, face, and predictive validity and has high translational and ethological value [39]. CSD has been well established as an animal stress model with a robust outcome: a depression-like phenotype with prominent anxiety, anhedonia, and social avoidance behaviors [40].

The present study aims at investigating the role of the eCB system in regulating the response to chronic stress. To this end, we employed a Targeted Recombination in Active Populations (TRAP) strategy to enable a permanent ensemble-specific KO of *Napepld*. The TRAP approach is commonly used for mapping physiological responses of defined neuronal populations using activity-dependent gene expression as a guide for Cre recombination [41–44]. Here, we analysed the effect of NAPE-PLD deletion in neurons active during the last three days of CSD stress on the phenotype of mice using a battery of behavioral tests. To increase the translational value of this research we applied a “therapeutic” approach and focused on the adaptation phase of chronic stress, instead of the reactive phase.

## Materials and methods

### Animals

All experiments were performed according to the European Community’s Council Directive of 22 September 2010 (2010/63EU) and approved by the respective agency (Landesuntersuchungsamt) of the State Rhineland-Palatinate (registration number G-17-1-005). Male mice were group-housed in temperature- and humidity-controlled rooms with a 12 h light-dark cycle with water and food provided *ad libitum*. Seven days prior to behavioral experiments, animals were single-housed. Mice used in this study were 8 weeks old by the start of the experiments.

We used transgenic mice derived from crossing B6.Cg-Tg(Arc-cre/ERT2)MRhn/CdnyJ (JAX Nr. 022357), called Arc-Cre here for simplicity [42], with NAPE-PLD-floxed mice [7], further referred to as Arc-NAPE-PLD line. Arc-Cre mice express Cre-ERT2 (a Cre recombinase, fused to a mutated human estrogen receptor ligand binding domain) under the gene-regulatory elements of the activity regulated cytoskeletal-associated protein (Arc). Arc-NAPE-PLD are lacking the NAPE-PLD enzyme specifically on neurons, expressing Arc, in the presence of tamoxifen (TAM, a synthetic estrogen receptor ligand). Arc-Cre^tg/wt^ x NAPE-PLD^fl/fl^ mice will be referred to as KO and compared to control littermate Arc-Cre^wt/wt^ x NAPE-PLD^fl/fl^ mice referred to as WT. Genotyping was performed before and after experiments.

### Chronic social defeat

Mice used in this study (both KO and WT) were exposed to CSD, which was carried out as previously reported [45]. Briefly, Arc-NAPE-PLD and adult retired breeder CD1 mice (Charles River) were housed in the same cage but were physically separated by a grid for 14 days. During this period, the separating grid was removed every day for 2 min per day to allow mouse interaction and the occurrence of multiple defeat episodes of attack from CD1 mice. After 14 days of CSD, mice were allowed to rest for one week (no exposure to CD1, single housing in home cage) before starting the behavioral tests.

### Tamoxifen injection

Arc-Cre-ERT2 is only active in the presence of its ligand TAM. To induce Cre-dependent recombination, Arc-NAPE-PLD KO, as well as control WT mice, were injected with TAM (Sigma Aldrich, Germany) intra peritoneally (i.p.) at a concentration of 20 mg/ml in a volume of 100 µl per mouse. TAM was dissolved in corn oil and ethanol (9:1 ratio, Sigma Aldrich), immediately frozen in aliquots, which were thawed at room temperature only once, mixed by inversion, and checked for precipitates before injection. Notably, TAM administration was performed on the last three days of CSD to trigger deletion of the AEA synthetizing NAPE-PLD in Arc-expressing neurons at a specific time-point (after/during stress exposure in the adaptation phase of CSD).

### Behavioral assays

Behavioral assessments were carried out during the light phase and trials were video-recorded and analyzed with Ethovision XT software, version 13 (Noldus, the Netherlands). After each trial the set-ups were cleaned with water.

#### The social interaction test (SI)

SI followed the procedure reported by [45] and was used to assess social behavior. Briefly, experimental animals were placed into an open field box (40 × 27 x 40 cm) with a small circular enclosure at one wall of the box and allowed to explore the arena for 2.5 min and were then placed back to their home-cage. After introducing the social target – naïve CD1 retired breeder mouse (target) – into the enclosure, the experimental animal was re-introduced to the box and allowed to explore for another 2.5 min. Time in the interaction zone, defined as a circular zone 2.5 cm in diameter around the enclosure, was recorded automatically with tracking software. The time the experimental animal spent in the interaction zone when the target mouse was absent (enclosure empty) was compared to the time spent in the interaction zone when the target mouse was present. SI ratio was calculated as 100 x (time in the interaction zone with a target mouse present) / (time in the interaction zone with target absent).

#### Light/dark test (LDT)

LDT was carried out as previously reported [46] and used to test anxiety-like behavior. Briefly, experimental animals were placed into a custom box (39 × 39 cm), divided into a light zone (two thirds of the box, white walls) and a dark zone (one third of the box, separated and covered by a 26 cm high lid, black walls). The light and dark compartments were connected by a small entry zone (5 ×5 cm). Animals were allowed to explore freely and move between the dark and the light zone for 6 min. Time spent in the light zone was assessed using video recording and subsequent automated analysis.

#### The elevated plus maze (EPM)

EPM was performed to assess anxiety-like behavior as previously reported [46], using a custom-made cross-shaped set-up having two open and two closed arms, elevated 100 cm above the floor. The arms of the maze were 35 cm long and 6 cm wide. Experimental mice were placed into the center of the maze facing the closed arms and were allowed to freely explore for 10 min. Animals were video-recorded and tracked automatically.

#### The tail suspension test (TST)

TST to measure emotional despair or depressive-like behavior followed the original reported procedure with modifications [47]. Mice were fixated by the tail onto a bright illuminated screen with fixative tape and left hanging for 6 min. The experimental animal was visually isolated from nearest objects. The mouse was recorded using a video-camera. Immobility was assessed automatically using tracking software.

#### Nesting behavior test (Nesting)

Nesting was performed and scored according to a previously established procedure [48] and was used to evaluate global well-being in mice and monitor whether CSD affects routinely performed tasks and shelter-seeking behavior. Briefly, a pad of compressed cotton was placed overnight into the home cage of the mouse. The following morning the quality and complexity of the nest were evaluated. A combined score (1 – no nest, 5 – perfect nest) was assigned for each mouse, combining the complexity of nest building and the amount of cotton that was left not processed.

### RNA and lipid analysis

#### Tissue extraction and processing

Three days after the last behavioral test, mice were deeply anesthetized using isoflurane (Abbott, USA) and decapitated. Trunk blood was collected in EDTA-coated tubes and immediately centrifuged at 10,000 rpm for 12 min at 4 °C. Serum was transferred to new tubes and frozen on dry ice. Brains were dissected and snap-frozen on a metal plate over dry ice. Tissue was stored at −80 °C until further processing.

#### RNA and lipid co-extraction

RNA and lipids were simultaneously isolated from the same tissue samples according to a recently published method for dual extraction of RNA and lipids from brain tissue [49]. Briefly, RNA was extracted by using the RNeasy® mini kit (Qiagen, Germany) according to standard procedure after adding a spike solution containing 10 µl internal standard (ISTDs) mixture of phospholipids, endocannabinoids, and chloroform to the samples to allow subsequent lipid isolation.

#### Reverse transcription and real-time quantitative PCR (RT-qPCR)

RNA (~ 500 ng per sample) was converted in cDNA by reverse transcription using the high-capacity cDNA reverse transcription kit (Applied Biosystems/Life Technologies, Germany). The cDNA (100 ng per reaction) was amplified by qPCR using the TaqMan Gene Expression Mastermix (Applied Biosystems/Life Technologies, Germany) and FAM dye-labelled TaqMan probes targeting the following genes: N-acyl phosphatidylethanolamine phospholipase D (*Napepld*) Mm00724596_m1; activity-regulated cytoskeleton-associated protein (*Arc*) Mm01204954_g1; FBJ osteosarcoma oncogene (*Fos*) Mm00487425_m1, Mm01302932_g1 and Mm00487426_g1; early growth response 1 (*Egr1*) Mm00656724_m1; regulator of G-protein signaling (*Rgs2*), Mm00501385_m1; fatty acid amide hydrolase (*Faah*) Mm00515684_m1; cannabinoid type-1 receptor (*Cnr1*) Mm00432621_s1; brain-derived neurotrophic factor (*Bdnf*) Mm04230607_s1; neuropeptide Y (*Npy*) Mm03048253_m1; FK506 binding protein 5 (*Fkbp5*) Mm00487406_m1; RAS-related C3 botulinum substrate 1 (*Rac1*) Mm01201653_mH; phosphodiesterase 11 A (*Pde11a*) Mm01327347_m1; and, as reference genes, glyceraldehyde-3-phosphate dehydrogenase (*Gapdh*) Mm99999915_g1. Data were analysed with an ABI 7300 real-time PCR cycler (Applied Biosystems/Life Technologies, Germany).

#### Liquid chromatography/mass spectrometry (LC/MS)

Lipid profiling was carried by using a SCIEX 5500 QTrap triple-quadrupole linear ion trap mass spectrometer (Concord, ON, Canada). LC conditions for eCBs and phospholipids (PLs) measurements followed in detail the procedure described by Post et al. [49]. The calibration standards N-arachidonoyl ethanolamine (AEA), 2-arachidonoyl glycerol (2-AG), arachidonic acid (AA), palmitoyl ethanolamide (PEA), and corresponding internal standards (ISTDs) (AEA-d4, 2-AG-d5, AA-d8, OEA-d2, PEA-d4 and 1-AG-d5) were purchased from BIOMOL Research Laboratories Inc. The calibration standards and corresponding ISTDs for phosphatidylethanolamine (PE 17:0–14:1) and phosphatidylcholine (PC 17:0–14:1) were purchased from Avanti Polar Lipids, Inc. Calibration curves were used for quantification of all target lipids using the MultiQuant 3.0. Software (AB SCIEX).

### Electrophysiological experiments

#### Modified social defeat stress

Arc-NAPE-PLD KO and WT female and male mice were subjected to modified social defeat stress (mSDS). Animals were placed into the cage of a retired breeder CD1 mouse 3 times for 5 min each. Between the 5 min exposure episodes, animals were separated by a metal mesh for 15 min. After the last exposure episode, the Arc-NAPE-PLD animals were placed back into their home-cage. This procedure was repeated on 5 consecutive days. TAM was injected as previously described during the last 3 days of stress 1 h prior to first exposure episode. After the stress, animals were allowed to rest in their home cages for 7 days prior to sacrifice.

#### Brain slice preparation

Brain slices were prepared from postnatal days (P) 50 to P71 in WT and KO mice. The animals were deeply anesthetized with isoflurane (Abbott, USA) and decapitated. The brain was rapidly removed and placed into ice-cold protective artificial cerebrospinal fluid containing (in mM): 110 choline chloride, 2.5 KCl, 1.25 NaH2PO_4_, 25 NaHCO_3_, 20 glucose, 11.6 sodium L-ascorbate, 3.1 sodium pyruvate, 0.2 CaCl_2_, 5 MgCl_2_. The solution was continuously bubbled with carbogen (95% O_2_ and 5% CO_2_), pH 7.4. Coronal slices of 300 μm thickness containing the prelimbic region of the mPFC [50] were prepared using a vibratome (Campden Instruments Ltd., UK). Slices were transferred into a storage chamber filled with artificial cerebrospinal fluid (aCSF) with a composition of (in mM): 126 NaCl, 2.5 KCl, 10 glucose, 1.25 NaH_2_PO_4_, 25 NaHCO_3_, 2 CaCl_2_, 1 MgCl_2_, continuously bubbled with carbogen, pH 7.4. Slices were stored for at least 60 min prior to recording.

#### MEA recordings and analysis

Extracellular recordings were performed with microelectrode arrays (MEA) consisting of 120 planar titanium nitrite electrodes with 4 internal references (120MEA100/30iR-Ti-pr, Multi-Channel Systems, Germany) using a MEA2100-System (Multi Channel Systems, Germany). Under a stereomicroscope, slices were carefully arranged onto MEAs in order to place the mPFC in correspondence with the field of electrodes. During recordings, slices were perfused with aCSF (equilibrated with 95% O_2_ / 5% CO_2_) at a rate of 3 ml/min through a peristaltic perfusion system and kept at 32 °C with a TC02 temperature controller connected to a heating plate and to a PH01 heatable perfusion cannula (Multi Channel Systems, Germany). Electrophysiological recordings were carried out for 10 min. Raw data from 120 channels were acquired at 50 kHz using MC_Rack software (Multi Channel Systems, Germany) and down-sampled to 200 Hz for local field potential (LFP) analysis. For LFP events detection, a threshold of 7 times the standard deviation of the noise was set on the negative slope of the signal and waveforms of max. 1100 ms were stored. Extracted datasets from all channels were then imported into Matlab 7.7 (Mathworks, MA, USA) for analysis of LFP event rates using a custom written routine. Only channels showing at least 4 LFP events in 10 min recordings were taken in consideration and counted as active. For comparisons, LFP rates were pooled across all recordings.

#### Whole-cell patch-clamp recordings and analysis

Brain slices were placed into a recording chamber (~0.7 ml volume) on the microscope stage (Axioscope FS, Zeiss, Germany). Slices were submerged with a constant flow of carbogenated aCSF. Flow rate was set to 1–1.5 ml/min. All experiments were performed at 31–32 °C. Pyramidal neurons in layers 2/3 of mPFC were selected visually according to morphological criteria. A 40x objective (Zeiss, Germany) was used. Patch pipettes were prepared from borosilicate glass capillaries using P-87 puller (Sutter Instrument Co., USA) and filled with an intracellular fluid (ICF) composed of (in mM): 130 KCl, 5 NaCl, 5 EGTA, 25 HEPES, 0.5 CaCl_2_, 2 Mg-ATP, 0.3 Na-GTP. pH was adjusted to 7.3 with KOH. Pipette resistance was 3–6 MOhms when filled with intracellular solution. Electrophysiological signals were acquired using an EPC-10 amplifier and TIDA 5.24 software (both from HEKA Elektronik, Germany). The signals were filtered at 3 kHz and sampled at a rate of 10 kHz. Liquid junction potential (<5 mV) was not corrected. Cells were patched in whole-cell configuration and the holding potential was set to −70 mV. Hyperpolarizing pulses of 10 mV were used to access cell capacity, series and access resistance. Only recordings with series resistance below 30 MOhm were accepted. Series resistance compensation was not applied. Cells exhibiting more than 20% changes in access resistance during an experiment were discarded. Action potentials (APs) were recorded in current clamp mode. Suprathreshold depolarizing stimuli of varying increasing amplitudes (500 ms) were applied to elicit APs. Miniature post-synaptic currents (mPSCs), both excitatory (mEPSCs) and inhibitory (mIPSCs), were recorded in presence of 0.5 μM tetrodotoxin (TTX), a blocker of voltage-gated sodium channels. mEPSCs were recorded in aCSF supplemented with 10 μM gabazine (Sigma, Germany), a blocker of γ-aminobutyric acid (GABA)_A_ receptors, and 50 μM DL-2-amino-5-phosphonopentanoic acid (DL-APV), a N-methyl-D-aspartic acid (NMDA) receptor blocker. mIPSCs were recorded in the presence of 10 μM 6,7-dinitroquinoxaline-2,3-dione (DNQX), an α-amino-3-hydroxy-5-methyl-4-isoxazolepropionic acid (AMPA)/kainate receptors antagonist, and 50 μM DL-APV. All drugs were purchased from BioTrend (Germany) unless otherwise specified. Evoked inhibitory postsynaptic currents (eIPSCs) were recorded in the presence of 10 µM DNQX and 50 µM DL-APV. Stimulation electrodes were prepared using the same method as for patch pipettes but filled with aCSF. Responses were evoked using focal electrical stimulation in the vicinity of the patched cell (100–150 µm laterally) using rectangular current pulses. The latter were delivered using a custom-built stimulation unit controlled by the amplifier. The intensity of the stimulation was regulated in order to elicit a unitary synaptic input (minimal stimulation). Electrophysiological data were evaluated using TIDA 5.24 (HEKA Elektronik, Germany). mPSCs were analyzed using PeakCount V3 software. The program employs a derivative threshold-crossing algorithm to detect individual PSCs. Each automatically detected event was displayed for visual inspection.

### Data analysis

#### Analysis of behavior testing, RNA and lipid analysis and electrophysiological experiments

No formal sample size estimation or randomization was performed, and all available KO mice and WT littermates were included in the experiments. Investigators were not blinded to allocation and outcome analysis. Data are represented as the mean±standard error of the mean (SEM). Statistical analysis was performed using GraphPad Prism 6 (GraphPad Software, CA, USA). D’Agostino-Pearson normality test was performed for all groups. Unpaired two-tailed Student’s t-test was used to analyze normally distributed data. Mann-Whitney test was performed in case of not normally distributed data. One or two-way analysis of variance (ANOVA) with Tukey’s or Bonferroni multiple comparison post-hoc test was performed where applicable. *p* < 0.05 was set as value to determine statistical significance. All reported p-values are two-tailed. Statistical significance was presented using the following rules: ^*^p < 0.05, ^**^p < 0.005, ^***^p < 0.001, ns- not significant.

#### Multivariate analysis

Multivariate analyses were performed using the R language and environment for statistical computing version 4.0.2 [51]. RNA expression and lipid data were first screened for outliers by transforming raw values to z-scores. In case that a z-score exceeded the critical threshold of |3.29 | (*p* < 0.001, two-sided z-test), the corresponding measurement was marked as an outlier and excluded from univariate statistical analysis (linear mixed effects models). Outliers were treated as missing values in the multivariate analyses. Missing values were imputed using the predictive mean matching method from the mice R package v3.10.0 with 10 multiple imputations. Molecular markers from different brain regions were represented in reduced multivariate space using principal component analysis (PCA) as implemented in the stats R package v4.0.2. Redundancy analysis (RDA) followed by permutational analysis of variance was performed to test the multivariate hypothesis if RNA expression and lipid data differ between brain regions and genotypes using the vegan R package v2.5–6. Sparse partial least squares discriminant analysis (sPLS-DA) models were calculated using the mixOmics package v6.12.1. Correlational analysis was performed by calculating pairwise Pearson correlation coefficients. Correlation matrices were visualized using the corrplot package v0.84. To identify important molecular features which were most predictive of key behavioral outcome measures in the EPM, LDT and SI tests, we fit multiple linear regression models with a lasso penalty for the maximum likelihood estimates using glmnet v4.1-3. The shrinkage penalty λ was estimated using 10-fold cross-validation implemented in the cv.glmnet function. The λ value associated with the smallest mean cross-validated error was chosen for the final penalized regression model. Univariate differences in RNA expression and lipid data between different brain regions and genotypes were investigated using linear mixed effects models as implemented in the lme4 package v1.1–23. Genotype and brain region were included as fixed effects in the model and animal as a random effect. Pairwise comparisons of model means were calculated using emmeans v1.4.8.

## Results

### Mice devoid of NAPE-PLD in neurons TRAPed at the last three days of CSD exhibit an anxiety-like phenotype

**Fig. 1:**
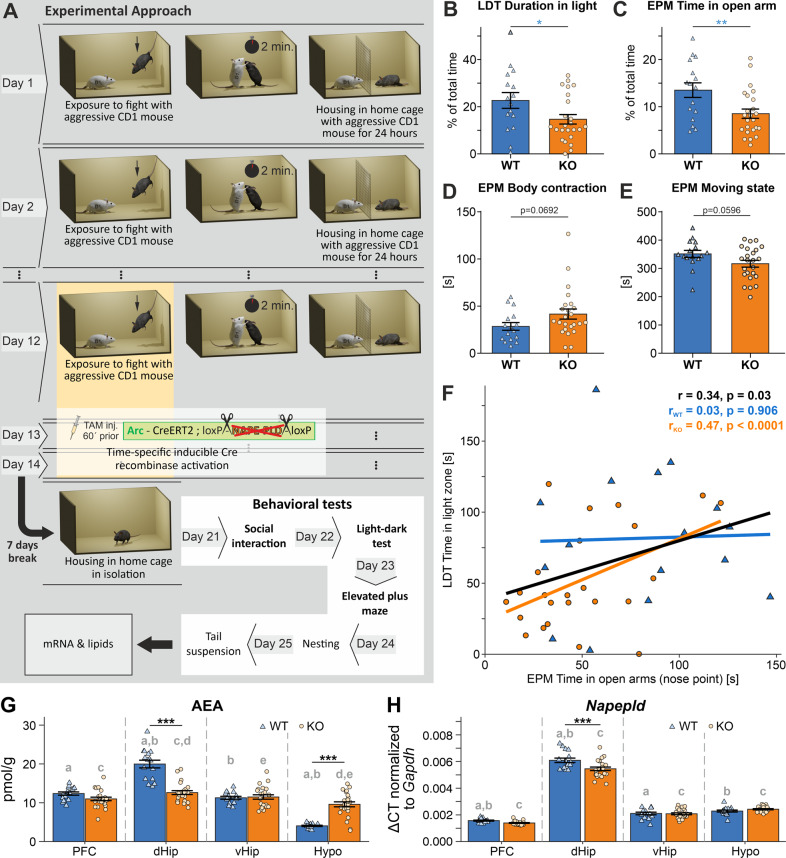
Anxiety-like phenotype of Arc-NAPE-PLD KO mice. **A** Scheme of experimental approach. CSD stress lasted for 14 days, TAM injections were performed on the last three days prior to CSD. The genetic manipulation in TRAPed neurons is illustrated: in Arc-expressing neurons, upon TAM application, the TAM-inducible CreERT2 recombinase excises NAPE-PLD gene sequences that are flanked by loxP sites. After seven days of housing in the home cage, behavioral tests were performed on day 21 to 25, followed by sacrifice, and mRNA and lipids extraction. **B–E** Group comparisons of Arc-NAPE-PLD WT and KO in standard behavioral tests. LDT: light-dark test (LDT), and elevated plus maze (EPM). Unpaired *t* test was used to identify significant differences, ^*^p<0.05, ^**^p<0.01. Data are represented as mean ± SEM. **F** Regression lines were drawn irrespective of genotype (black) as well as separately for WT (blue; *n* = 16) and KO (orange; *n* = 24) animals. The overall Pearson correlation coefficient as well as the correlation coefficients for each genotype and the respective *p*-value are shown on the plots. **G, H** AEA and *Napepld* mRNA levels in brain regions of stressed Arc-NAPE-PLD WT and KO mice. **G** AEA levels were measured using LC/MS. **H** *Napepld* mRNA levels were normalized to *Gapdh*. Genotype and brain region differences were assessed using linear mixed effects models. Genotype and brain region were included as fixed effects in the model and animal as random effect. Pairwise comparisons were based on estimated model means with Tukey *p* value adjustment in case of multiple comparisons. Asterisks indicate significant differences between genotypes in a specific region: ^***^ *p* < 0.001. Letters indicate the significance of pairwise comparisons of different brain regions within the same genotype. Groups sharing the same letter are significantly different from each other at *p* < 0.05. Data are represented as mean ± SEM and individual values; WT/KO *n* = 16/24. PFC prefrontal cortex, dHip dorsal hippocampus, vHip ventral hippocampus, Hypo hypothalamus, Gapdh glyceraldehyde-3-phosphate-dehydrogenase, LC/MS liquid chromatography/mass spectrometry.

Arc-NAPE-PLD WT and KO animals were injected with TAM i.p. every day during the last three days of the CSD protocol (day 12–14), one hour before the defeat episode (Fig. 1A). We observed a pronounced anxiety-like phenotype in stressed Arc-NAPE-PLD KO mice in LDT and EPM. Mice spent significantly less time in the light compartment of the LDT (Fig. 1B, *p* = 0.0443). In the EPM, KO mice spent less time in the open arms of the apparatus than WT (Fig. 1C, *p* = 0.0070). Furthermore, KO mice showed a tendency of keeping their body longer in a contracted position than WT (Fig. 1D, *p* = 0.0692) as well as spending more time not moving (Fig. 1E, *p* = 0.0596), indicating a decrease in exploration behavior, which is a manifestation of an anxiety-like state. The time spent in the open arms of the EPM and in the light compartment in the LDT test were positively correlated when genotypes were pooled together (Fig. 1F), pointing to the coherent anxiety-like phenotype and validity of the behavioral tests. This effect was even more pronounced in the KO group. There were no differences found in other behavioral tests such as SI (*p* = 0.8336), Nesting (*p* = 0.8082), and TST (0.5225) between Arc-NAPE-PLD WT and KO, as well as no differences in bodyweight during the CSD and at the beginning of behavioral testing (Supplementary Fig. 1). In a separate experiment, we compared control non-stressed Arc-NAPE-PLD WT and KO mice that were single-housed for 12 days and then injected with TAM i.p. for 3 consecutive days (equivalent to day 12–14 of CSD). 7 days after the last TAM injection, the battery of behavioral tests was performed. We did not observe any genotype effects in these non-stressed mice (Supplementary Fig. 2).

### TRAP recombination leads to decreased *Napepld* expression and reduced AEA content in selected brain regions of Arc-NAPE-PLD KO mice

The observed phenotype of Arc-NAPE-PLD KO mice, exposed to CSD and TRAPed during the last three days of the stress paradigm, led us to investigate the molecular drivers of the anxiety-like behavior. WT and KO mice were sacrificed two days after the last behavioral test; brain tissue was collected and immediately frozen. The right hemisphere was manually dissected into the prefrontal cortex (PFC), dorsal and ventral hippocampus (dHip and vHip, respectively), and the hypothalamus (Hypo). The tissue was later processed in the dual extraction procedure [49] to obtain the lipid fraction and the RNA fraction.

We validated the TRAP recombination by measuring the AEA content and *Napepld* mRNA in the brains of stressed Arc-NAPE-PLD KO and stressed littermate WT mice. We observed a significant decrease of AEA in the dHip of KO mice (Fig. 1G, *p* < 0.001) compared to WT. The same trend was present in the PFC, but the difference was not statistically significant (*p* = 0.09). Paradoxically, there was an increase of AEA in the Hypo of the KO mice compared to WT. We also detected a significant decrease of PEA in dHip and vHip of KO mice (Supplementary Fig. 3A), as well as an increase of 2-AG in dHip and a decrease in Hypo of KO mice (Supplementary Fig. 3B). PEA is a N-acylethanolamines and similarly to AEA it is synthesized by NAPE-PLD, among other enzymes. The mRNA levels of *Napepld* were significantly decreased in the dHip of KO compared to WT (Fig. 1H, *p* < 0.001). No changes of *Napepld* expression were observed in the PFC, vHip, and Hypo of KO animals compared to the WT group. Herewith we confirmed the TRAPing of a subset of neurons, mostly in the dHip and partially in PFC.

### Distinct molecular signatures of selected brain regions of Arc-NAPE-PLD KO mice

**Fig. 2:**
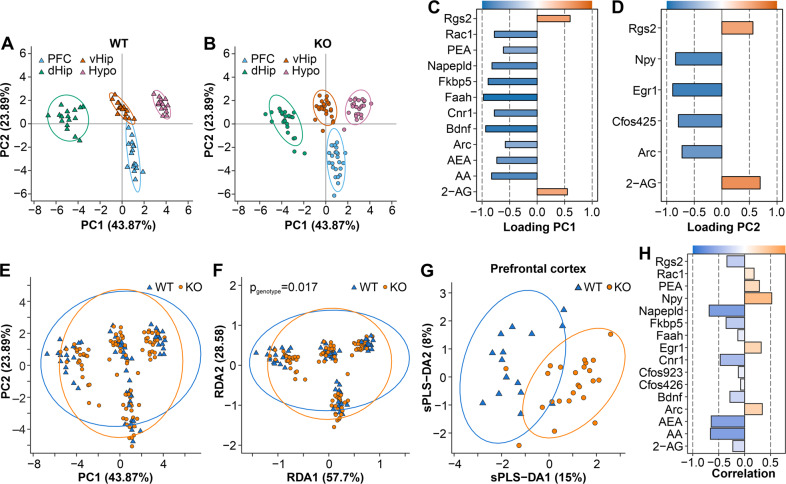
Principal component analysis (PCA) of endocannabinoids, arachidonic acid and analysed transcripts in different brain regions of stressed Arc-NAPE-PLD WT and KO mice. The PCA model was calculated using all data from WT and KO mice. After the ordination procedure, WT **(A)** and KO **(B)** animals were visualized separately to investigate differences between brain regions within each genotype. The percentage of variance explained by each dimension is indicated on the respective axis in **A** and **B**. Confidence ellipses around each brain region were drawn at the 95% level. Loadings of the original variables on the first (PC1) and second principal component (PC2) are shown in **C** and **D**, respectively. Variables with an absolute loading greater than 0.4 were considered to significantly contribute to the observed multivariate pattern. **E–H** Multivariate differences in endocannabinoids, arachidonic acid and mRNA levels between stressed Arc-NAPE-PLD WT and KO mice. The effect of genotype for all brain regions was investigated using PCA or RDA. **G** The impact of genotype on the RDA ordination was examined statistically using permutational analysis of variance. Multivariate differences between WT and KO mice in the PFC were evaluated additionally using sPLS-DA. The percentage of variance explained by each dimension is indicated on the respective axis in **E–G**. Confidence ellipses for each genotype were drawn at the 95% level. **H** Correlations of the original variables with the first multivariate dimension (sPLS-DA1).

**Fig. 3:**
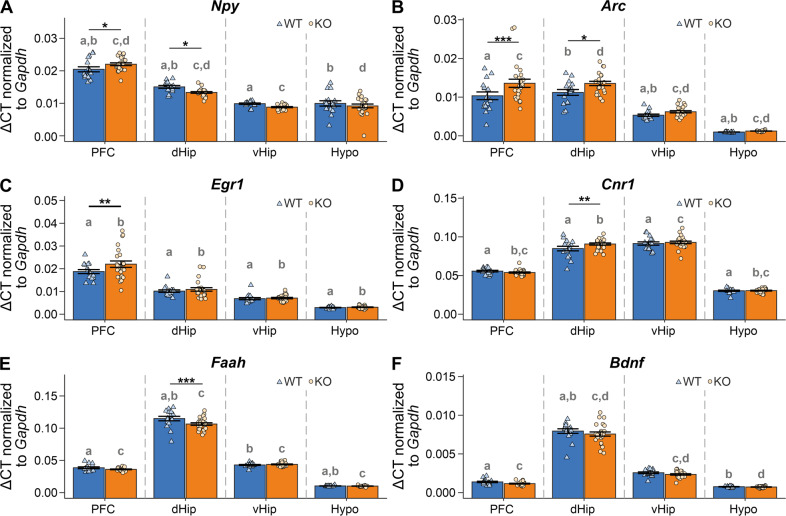
mRNA levels of selected molecular targets in brain regions of stressed Arc-NAPE-PLD WT and KO mice. **A** *Npy* mRNA levels, **B** *Arc* mRNA levels, **C** *Egr1* mRNA levels, **D** *Cnr1* mRNA levels, **E** *Faah* mRNA levels, **F** *Bdnf* mRNA levels; mRNA levels were normalized to *Gapdh*. Genotype and brain region differences were assessed using linear mixed-effects models. Genotype and brain region were included as fixed effects in the model and animal as random effect. Pairwise comparisons were based on estimated model means with Tukey *p*-value adjustment in case of multiple comparisons. Asterisks indicate significant differences between genotypes in a specific region: ^*^p < 0.05; ^**^p < 0.01; ^***^p < 0.001. Letters indicate the significance of pairwise comparisons of different brain regions within the same genotype. Groups sharing the same letter are significantly different from each other at *p* ≤ 0.05. Data are represented as mean ± SEM and individual values; WT/KO n = 16/24. Npy neuropeptide Y, Erg1 early growth response factor 1, Cnr1 cannabinoid CB1 receptor, Faah fatty acid hydrolase, Bdnf brain-derived neurotrophic factor.

Next, we asked whether the different brain regions of stressed Arc-NAPE-PLD WT and KO mice were associated with distinct molecular signatures based on the selected eCBs and eCB-related lipids: AEA, 2-AG, arachidonic acid (AA), palmitoyl ethanolamide (PEA), and mRNA markers: *Napepld*; activity-regulated cytoskeleton-associated protein (*Arc*); FBJ osteosarcoma oncogene (*Fos*); early growth response factor 1 (*Egr1*); regulator of G-protein signaling (*Rgs2*); fatty acid amide hydrolase (*Faah)*; *Cnr1*; brain-derived neurotrophic factor (*Bdnf*); neuropeptide Y (*Npy*); FK506 binding protein 5 (*Fkbp5*); RAS-related C3 botulinum substrate 1 (*Rac1*). To this end, we performed a principal component analysis (PCA), revealing that each brain region formed a distinct cluster (Fig. 2A, B). dHip was separated from PFC/vHip and Hypo along the first multivariate dimension (PC1), which captured 43.87% of the total variance. While the pattern was similar for WT and KO mice, differences between brain regions were more pronounced for WT animals, as indicated by the brain clusters for KO mice appearing closer to each other in the reduced multivariate space (Fig. 2B). To evaluate which of the original variables included in the analysis can explain this multivariate pattern, we calculated the loadings on PC1, which correspond to correlations of the original variables with the multivariate dimension (Fig. 2C). Altogether, 12 out of the 15 investigated molecular markers significantly contributed to differences between the brain regions. *Rgs2* (controls signaling through G-protein coupled receptors) and the eCB 2-AG were positively correlated with PC1, indicating that, on average, the levels of these markers were highest in the Hypo. All additional markers were negatively correlated with PC1. Furthermore, we calculated the loadings on PC2, which explained the differences between the PFC and vHip (Fig. 2D). Six out of the 15 molecular markers significantly contributed to this pattern. 2-AG and *Rgs2* were again positively correlated with PC2, pointing to increased levels in vHip compared to PFC. Apart from the differences between brain regions, we were also interested in elucidating the impact of genotype on the multivariate molecular signature. We did not observe a distinct separation between genotypes in the PCA analysis (Fig. 2E). Therefore, we employed redundancy analysis (RDA), which is the constrained version of PCA. We included genotype and brain region as well as their interaction as explanatory variables in the ordination procedure. WT mice were not visibly separated from KO animals on the RDA ordination plot (Fig. 2F). However, permutational analysis of variance revealed that the effects of genotype (*p* = 0.017) and the interaction term genotype/brain region (*p* = 0.001) were statistically significant. To more specifically investigate genotype differences, we performed sparse partial least squares discriminant analyses (sPLS-DA) for each brain region separately. PLS-DA offers higher sensitivity for detecting group differences compared to PCA and RDA while simultaneously being a robust method against false positives [52]. Results from this analysis for the PFC are shown in Fig. 2G. KO animals were separated from the WT littermates along the first multivariate dimension and the important molecular features explaining this pattern were *Napepld*, AEA, AA, and *Cnr1*, which on average had higher levels in WT animals as well as *Npy*, *Egr1*, and *Arc*, which were associated with increased levels in KO mice (Fig. 2H). To confirm these patterns we detected with the multivariate techniques, we also performed univariate analyses investigating the impact of brain region and genotype on specific molecular targets (Fig. 3).

There was a significant increase of *Npy* in the PFC and a decrease in dHip of KO mice compared to WT. Additionally, we observed high variability of expression patterns of *Npy* across the analyzed brain regions with the highest expression in PFC and the lowest in vHip (Fig. 3A). To our surprise, the expression of the immediate-early gene (IEG) *Arc* was increased in PFC (*p* = 0.0006) and dHip (*p* = 0.013) of KO animals as compared to WT (Fig. 3B). These findings might indicate increased neuronal activity in these regions after CSD stress. Increased expression of another plasticity and activity-associated gene *Egr1* was detected in PFC (*p* = 0.0038) of KO mice, compared to WT (Fig. 3C). However, no changes between genotypes were found in other regions. Another protein associated with neuronal plasticity is BDNF. *Bdnf* mRNA was slightly downregulated in dHip of KO mice (Fig. 3F) but did not reach significance (*p* = 0.0865). The expression of *Cnr1* was significantly increased in dHip of KO mice (Fig. 3D, *p* = 0.0061), which might indicate the recruitment of CB1 following the abolishment of NAPE-PLD-mediated synthesis of AEA. Moreover, the *Faah* expression was significantly reduced in dHip of KO mice (Fig. 3E, *p* < 0.001) to potentially compensate for the decreased level of AEA in dHip (see Fig. 1G).

### Individual behavioral performance correlates with the brain’s molecular signature

Additionally, we were interested in elucidating the potential relationship between the molecular signature of individual mice and their performance in the behavioral tests. Therefore, we calculated the pairwise correlations between all molecular and behavioral outcome measures, which identified multiple potential associations between behavioral and molecular features (Supplementary Fig. 4).

**Fig. 4:**
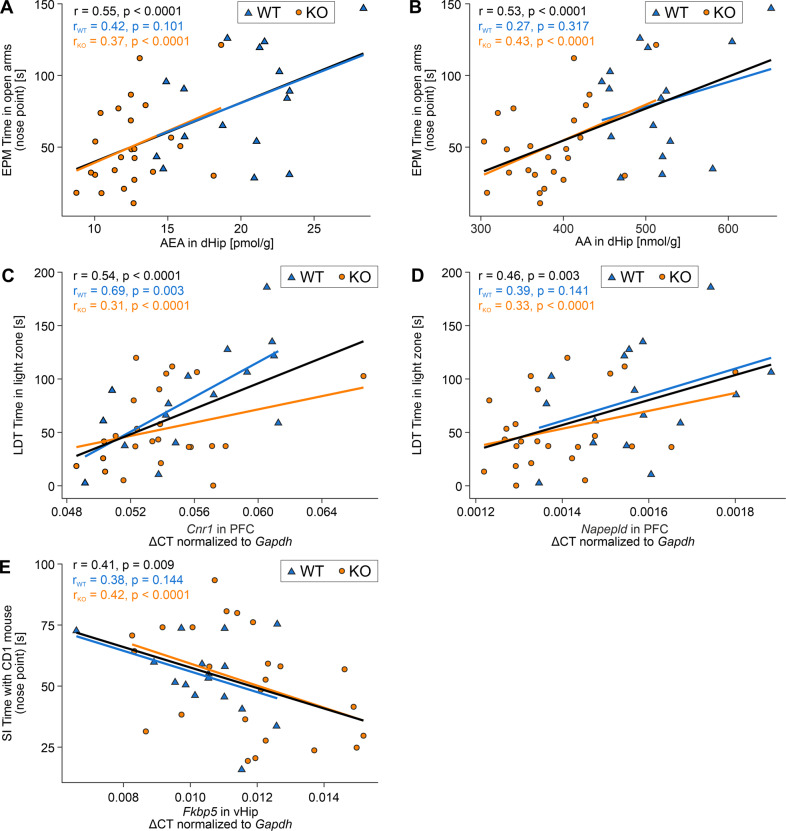
Correlation between selected behavioral response measurements and molecular markers in selected brain regions of Arc-NAPE-PLD WT and KO mice. **A, B** Correlation between AEA and AA levels in dHip and the time mice spent in the open arms of the EPM. **C, D** Correlation between *Cnr1* and *Napepld* mRNA levels in the PFC and the time mice spent in the light compartment of the LDT. **E** Correlation between *Fkbp5* mRNA levels in vHip and the time spent interacting with the CD1 mouse in the SI test. Regression lines were drawn irrespective of genotype (black) as well as separately for WT (blue; *n* = 16) and KO (orange; *n* = 24) animals. The overall Pearson correlation coefficient as well as the correlation coefficients for each genotype and the respective *p*-value are shown on the plots. Fkbp5 FK506 binding protein 5.

To limit the analysis to the most representative outputs of the behavioral tests, we focused on the time spent in the light compartment in the LDT and the time the nose point of the mouse was detected in the open arms of the EPM as a proxy of anxiety-like behavior. The time spent interacting with the CD1 mouse in the SI test (nose point detected in proximity to CD1 mouse) was used as a proxy for sociability and generalization after CSD. We performed lasso regression for each selected behavioral measure as response variable to identify important molecular markers, which had the highest predictive value for the performance in the respective behavioral test and could thereby serve as biomarkers of anxiety-like behavior and social avoidance. The lasso regularization method facilitates feature selection by setting the regression coefficients of covariates with low predictive value to 0. This analysis revealed that the AEA and AA levels in dHip were the most important molecular markers predictive of the time spent in the open arms. A higher concentration of both AEA and AA in dHip was significantly correlated with an increased duration of stay in the open arms when both genotypes were analyzed together (Fig. 4A, B). The same trend was also present for WT and KO animals when separately analyzed; however, the association was no longer significant in WT mice, likely due to small sample size.

Additionally, expression levels of *Cnr1* and *Napepld* in PFC were the most relevant variables for predicting the time spent in the light compartment of the LDT (Fig. 4C, D). Increased mRNA levels of *Cnr1* in the PFC of both KO and WT were associated with reduced anxiety behavior, as indicated by more time spent in the light compartment of the LDT (*r* = 0.54, *p* < 0.0001). This effect was even more pronounced in the WT subgroup (*r* = 0.69, *p* = 0.003). Increased *Napepld* expression in PFC was also positively correlated with a reduced anxiety-like phenotype. However, the association was no longer statistically significant when analyzing WT animals only. Finally, we identified *Fkbp5* expression levels in vHip as an important molecular marker that was significantly positively correlated with social behavior in the SI test (Fig. 4E). Increasing mRNA concentrations of *Fkbp5* were associated with more pronounced social avoidance indicated by less time spent interacting with the CD1 aggressor mouse.

### Electrophysiological profiling of the PFC of stressed Arc-NAPE-PLD KO mice

#### Large-scale network activity

To address the observed increase of *Arc* expression in the PFC (see Fig. 3B) as well as to characterize neurophysiological properties of the PFC of stressed mice, Arc-NAPE-PLD WT and KO female and male mice were subjected to a modified social defeat stress (mSDS), injected with TAM in the last three days of mSDS and sacrificed 7 days later. The effectiveness of the mSDS procedure to trigger Arc-mediated recombination was controlled by a genomic PCR performed on brain slices used for electrophysiological measurements (Supplementary Fig. 5).

**Fig. 5:**
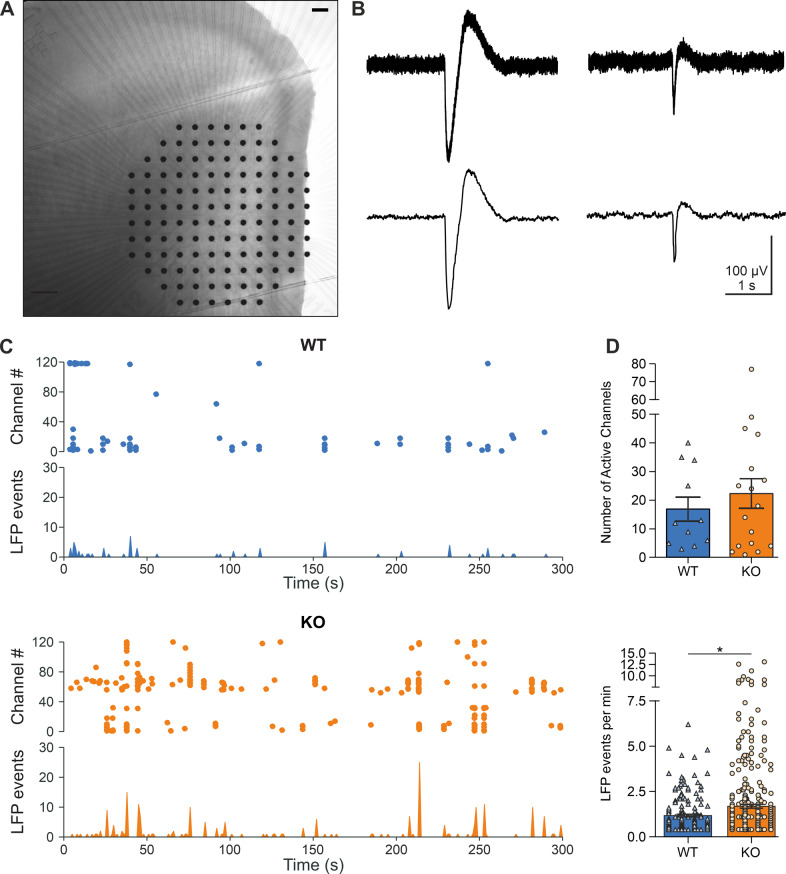
Large-scale network activity in prelimbic cortex of Arc-NAPE-PLD WT and KO mice. **A** Coronal slice of the prelimbic region of the mPFC on a MEA; scale bar 100 µm. **B** Representative traces of two typical LFP events, raw traces (top) and low-pass filtered (bottom). **C** Representative raster plots (top) and frequency bar plots (bottom) of spontaneous LFP events in WT and KO animal. **D** Comparison of number of active channels (top) and mean LFP event rates (bottom) in slices from WT and KO mice. Statistical significance was evaluated using Mann–Whitney´s U test, ^*^p < 0.05. MEA microelectrode array, LFP local field potential.

Extracellular recordings of the local field potential (LFP) were performed in acute brain slices of the prelimbic region of the mPFC using a 120 channels microelectrode array (MEA) (Fig. 5A). Spontaneous LFP activity in slices from Arc-NAPE-PLD WT and KO animals was rather low and characterized by both isolated and synchronized events on multiple channels (Fig. 5C). Two types of LFP events were detected: the vast majority had a shorter (<500 ms) duration and lower amplitude, whereas the residual ones had a slower kinetic (~1 s) and larger amplitude (Fig. 5B). Slices from KO animals displayed a higher (22.4 ± 5.2, *n* = 17 slices), although not significant number of active channels than WT ones (16.9 ± 4.2, *n* = 11 slices) (Fig. 5C, D). The average frequency of LFP events was significantly (*p* < 0.05) higher in KO (1.69 ± 0.10 min^−1^, *n* = 380 channels) than in WT animals (1.17 ± 0.07 min^−1^, *n* = 186).

#### Intrinsic and synaptic properties

Pyramidal neurons in layer (L) 2/3 of the prelimbic region of the mPFC were recorded using whole cell patch clamp. Average membrane resistance was significantly (*p* < 0.05) higher in Arc-NAPE-PLD KO neurons (133.4 ± 4.4 MΩ, *n* = 54) as compared to WT (119.1 ± 4.2 MΩ, *n* = 38 (Supplementary Fig. 6B). In contrast, resting membrane potential (WT: −61 ± 0.5 mV; KO: −60.6 ± 0.4 mV, *p* = 0.61), membrane capacitance (WT: 32.9 ± 1.5 pF; KO: 30.8 ± 0.9 pF, *p* = 0.2) and membrane time constant (WT: 0.7 ± 0.02 ms; KO: 0.69 ± 0.02 ms, *p* = 0.85) were not significantly different between WT and KO (Supplementary Fig. 6A, C, D). Action potentials were significantly (*p* < 0.05) larger in KO cells (92.4 ± 0.8 mV, *n* = 22) than in WT neurons (89.6 ± 0.7 mV, *n* = 12) (Supplementary Fig. 6H). Other action potential properties such as threshold (WT: −35.5 ± 0.9 mV vs KO: −36.7 ± 0.8 mV, *p* = 0.31) and duration (WT: 3.54 ± 0.14 ms vs KO: 3.6 ± 0.13 ms, *p* = 0.77) were not different between WT and KO (Supplementary Fig. 6G, I). Depolarizing current pulse injections of increasing amplitude did not reveal any significant differences in firing frequencies in WT and KO (Supplementary Fig. 6E, F).

**Fig. 6:**
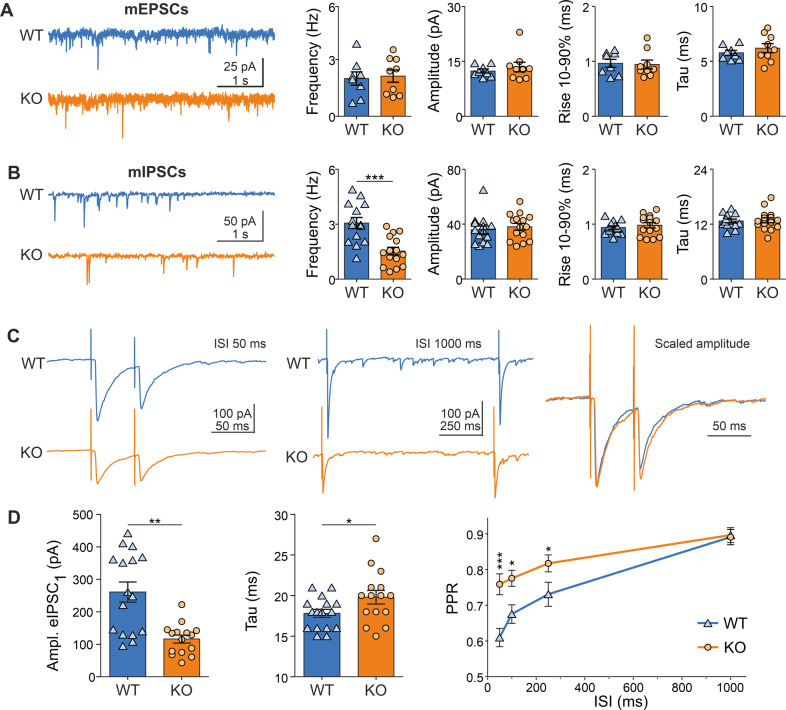
Miniature postsynaptic currents and evoked inhibitory postsynaptic currents in prelimbic cortical neurons of Arc-NAPE-PLD WT and KO mice. **A** Representative traces of miniature excitatory postsynaptic currents (mEPSCs) in neurons from WT and KO mice and analysis of mean frequency, amplitude, rise time and decay time in neurons from WT (*n* = 8) and KO (*n* = 9) mice. **B** Representative traces of miniatures inhibitory postsynaptic currents (mIPSCs) in neurons from WT (*n* = 14) and KO (*n* = 15) mice. The mean frequency of mIPSCs was significantly lower in KO animals than in WT (Student´s *t* test, ^***^p < 0.001). **C** Representative traces of evoked inhibitory postsynaptic currents at interstimulus interval (ISI) of 50 ms (left) and 1000 ms (center). Scaled events superimposed (right). Each trace is the average of 10 repetitions. **D** Statistical analysis demonstrated reduced amplitude of the mean first event as well as longer decay time in neurons from KO mice compared to WT. The paired-pulse ratio (PPR) was significantly increased in KO mice for ISI up to 250 ms (ANOVA, Bonferroni´s multiple comparisons test). Statistical significance was evaluated using Mann-Whitney´s U test for amplitude of first event and Student´s *t* test for decay time. ^*^p < 0.05, ^**^p < 0.005.

Miniature excitatory postsynaptic currents (mEPSCs) did not reveal any differences between WT and KO neurons in either frequency (WT: 2.06 ± 0.36 Hz vs KO: 2.19 ± 0.34 Hz, *p* = 0.79) or amplitude (WT: 12.36 ± 0.56 pA vs KO: 13.45 ± 1.31 pA, *p* = 0.48) (Fig. 6A). Rise time (WT: 0.96 ± 0.07 ms vs KO: 0.94 ± 0.08 ms, *p* = 0.86) and decay time of the events (WT: 5.77 ± 0.23 ms vs KO: 6.20 ± 0.42 ms, *p* = 0.4) were not significantly different in WT and KO neurons. While amplitude (WT: 35.86 ± 2.83 pA vs KO: 38.05 ± 2.49 pA, *p* = 0.29) and kinetic properties (rise time - WT: 0.94 ± 0.03 ms vs KO: 0.98 ± 0.05 ms, *p* = 0.48, decay time - WT: 12.70 ± 0.40 ms vs KO: 12.86 ± 0.57 ms) of miniature inhibitory postsynaptic currents (mIPSCs) were comparable in WT and KO neurons, their mean mIPSC frequency was significantly (*p* < 0.001) decreased in KO neurons (1.53 ± 0.21 Hz, *n* = 15) as compared to WT (3.066 ± 0.30 Hz, *n* = 14) (Fig. 6B). To elucidate the mechanisms underlying the lower frequency of mIPSCs in KO neurons, we recorded evoked inhibitory postsynaptic currents (eIPSCs).

Paired pulse ratio (PPR) was measured using four different interstimulus intervals (ISI): 50, 100, 250 and 1000 ms. The average PPR was significantly increased in KO neurons (Fig. 6C, D, F_7.116_ = 12.75, *p* < 0.0001, ANOVA), suggesting a decrease in release probability. In particular, the difference in PPR was larger (*p* < 0.0001, Bonferroni´s multiple comparisons post hoc test) at the shortest time interval, 0.59 ± 0.03 (*n* = 16) for WT and 0.79 ± 0.03 (*n* = 15) for KO at 50 ms ISI. This increase in PPR in KO neurons was present as well at ISI of 100 ms (WT: 0.66 ± 0.03 vs KO: 0.79 ± 0.02, *p* < 0.05, Bonferroni multiple comparisons test) and 250 ms (WT: 0.72 ± 0.04 vs KO: 0.83 ± 0.03, *p* < 0.05, Bonferroni multiple comparisons test). At the longest ISI of 1000 ms, the mean PPR did not differ between the two genotypes, as both displayed a PPR of 0.89 ± 0.03 ms. Another indication of reduced release probability in KO animals was the reduction (*p* < 0.005, Mann–Whitney´s U test) in amplitude of the first response in KO neurons (116.2 ± 12.24 pA, *n* = 15), as compared to WT (260.7 ± 30.92 pA, *n* = 16). Interestingly, the decay time was significantly slower (*p* < 0.05) in events from KO cells (19.80 ± 0.83 ms, *n* = 15) relative to responses recorded in WT neurons (17.81 ± 0.49 ms, −*n* = 16).

The measurements are consistent with Arc-NAPE-PLD KO animals having morphologically similar L2/3 pyramidal neurons (at least, the perisomatic size), with an increased membrane resistance. Moreover, the evoked APs show slightly increased amplitude. Those cells have a relatively similar composition of synaptic GABA_A_ receptors as WT neurons but receive a lower level of GABAergic synaptic inhibition. Investigation using evoked inhibitory responses suggests a decrease in release probability of inhibitory terminals on L2/3 pyramidal neurons.

## Discussion

In the current study, we leveraged a recently developed TRAP system to genetically manipulate only a subset of neurons that were active at a selected time point during social stress. This sophisticated experimental system allowed us to identify a pronounced anxiety-like phenotype in Arc-NAPE-PLD KO mice. By subsequently employing the dual lipid/mRNA extraction from target key brain regions of these mice, we examined crucial molecular components and targets of the eCB system and the stress response. Our results shed light into the potential mechanisms leading to an increased anxiogenic phenotype as a consequence of reduced AEA signaling in stress-activated neurons.

### Impairment of the negative feedback loop of the HPA axis

Glucocorticoid receptors (GR), the major mediators of the negative feedback regulation of the HPA axis, are abundantly expressed in the PFC, Hip, and Hypo. The interaction of the eCB system, in particular of the CB1, and glucocorticoid signaling arguably plays an important role in controlling the emotional, physiological, and adaptive responses to stress [53].

We observed a strong decrease of *Napepld* mRNA in the dHip of stressed Arc-NAPE-PLD KO mice and a concomitant decrease of AEA in this brain region as compared to stressed WT. The expression of *Faah* in dHip was downregulated, possibly as a compensatory mechanism for the decrease of AEA. Similarly, the expression of *Cnr1* was upregulated in dHip. These findings are in accordance with several stress studies on rodents, as discussed below.

Exposure to repeated stress, such as restraint and social defeat, has previously been reported to be associated with the reduction of AEA levels in the hippocampus [24]. The authors hypothesized that decreased AEA levels contributed to an increase in HPA axis activity and observed a negative correlation between the levels of AEA and basal CORT, a marker for HPA axix activity. Moreover, a high basal expression of *Cnr1* was found in Wistar Kyoto (WKY) rats, a model of depressive-like behavior [54]. The upregulation of CB1 is believed to be a compensatory mechanism in response to a reduced AEA signaling in the hippocampus of these rats, mediated by the elevation of basal FAAH activity. When treated with a FAAH inhibitor, URB597, the WKY rats displayed an increase in AEA and a decrease in manifestations of depressive-like behavior.

Interestingly, in the context of the glucocorticoid feedback loop, we also observed a negative correlation between FK506 binding protein 51 (*Fkbp5*) mRNA levels and the performance in the SI test across Arc-NAPE-PLD WT and KO mice, whereby the performance in the SI test is considered to be a behavioral correlate of stress resilience. Prolonged expression of FKBP5 following CORT release in response to a stressor leads to inhibition of the negative feedback loop, resulting in prolonged elevated circulation of CORT and consequent maladaptive stress response [55]. FKBP5 KO mice exhibit mild GR hypersensitivity and improved coping behavior after acute stress exposure [56]. Moreover, FKBP5 KO mice were shown to be less responsive to the deleterious effects of CSD stress in terms of behavioral and neuroendocrine responses [57]. Additionally, mice treated with a recent FKBP5 inhibitor during CSD show reduced social withdrawal and anxiety-like behavior [58], which is in line with our data.

Our findings suggest an impairment of the negative feedback loop after prolonged stress exposure in Arc-NAPE-PLD KO mice. To validate this hypothesis, it is necessary to include measurements of the basal activity (plasma levels of CORT) and the functional state of the HPA axis (HPA reactivity assay) in future experiments [59].

### Prefrontal cortex exhibits reduced control over amygdala

NPY is involved in stress processing; it is thought to be implicated in the termination of stress response and interaction with the HPA axis [60]. We observed an increase of the NPY in PFC of Arc-NAPE-PLD KO mice after CSD stress compared to WT. This finding is in agreement with a study where chronic variable stress induced an increase of NPY in the PFC, while reduced levels were observed in the amygdala [61]. An increase of NPY in cortical interneurons might dampen the inhibiting output of the PFC to the amygdala [62]. A reduction in NPY signaling in the amygdala leads to negative behavioral consequences, such as anxiety [63]. Therefore, reduced PFC control over amygdala may lead to a stress-susceptible phenotype. In future studies, amygdala should be integrated into the analysis of molecular targets to investigate this hypothesis in more detail.

### Neuroplasticity and stress response

In contrast to an increase of NPY in the PFC, we discovered a decrease of NPY mRNA in the dHip of stressed Arc-NAPE-PLD KO mice. This finding is in accordance with a study where mice, exposed to CUS and exhibiting a highly disrupted phenotype, displayed a reduction of NPY in several brain regions, such as the periaqueductal grey, the amygdala, and the hippocampus, compared to non-stressed controls. Furthermore, a single dose of NPY microinfused into the dHip of stressed mice one hour after stress exposure reduced manifestations of anxiety and avoidance behavior [64]. The microinjection of NPY was accompanied by an increase of BDNF. There is an anticipated connection between NPY and BDNF, i.e., TrkB (BDNF receptor) activity was shown to influence the expression of NPY in hippocampal slice cultures [65]. We observed a tendency of decreased BDNF levels in dHip of stressed Arc-NAPE-PLD KO compared to stressed WT. A reduction of BDNF in hippocampus was shown in response to stress [66], whereas an overexpression of BDNF played a stress-protective role [67]. It is hypothesized that increased CORT secretion due to HPA axis activity suppresses BDNF expression. These effects can be restored by prolonged antidepressant treatment [68].

In a recent study, a connection between the NPY and eCBs was established. NPY was shown to mediate anxiolytic and antidepressant effects of elevated AEA due to the action of URB597 in shock and reminders models of PTSD in rats. Moreover, the authors hypothesized that AEA is upstream of the effects of NPY, possibly modulating the sensitivity of the NPY receptor [69].

We observed an increase in the early growth response factor 1 (*Egr1*) mRNA levels in the PFC of stressed Arc-NAPE-PLD KO mice. EGR1 is an IEG involved in synaptic plasticity, neuronal activity, learning and memory, response to emotional stress, and reward [70]. Interestingly, there is a reported crosstalk between EGR1, BDNF, and GR. GR represses BDNF expression, acting on the activity-regulated BDNF transcript 4 by trans-repression on a DNA site for EGR1 binding, located on the transcription start site of transcript 4, thus establishing a direct functional interaction between GR and EGR1 [71].

To conclude, NPY, BDNF and EGR1 are interconnected factors important for neuronal plasticity. Deficits in synaptic plasticity in key regions such as hippocampus and PFC, responsible for emotional memory and stress processing, can impair the flexibility and adaptation capacity of neural circuits and form a substrate for the development of behavioral abnormalities and pathologies after chronic stress [72]. It is therefore important to further investigate the mechanisms of action of these and other genes involved in neuroplasticity as well as their interactions with other systems, such as the eCB system.

### Hyperexcited state of the PFC

It is important to mention that another IEG, *Arc*, was also upregulated in the PFC of KO mice, indicating a possible hyperexcited state. Using in vitro electrophysiological measurements of the prelimbic region of the mPFC of stressed Arc-NAPE-PLD WT and KO mice, we found differences in passive and active intrinsic membrane properties. The largest effect was a strong decrease in GABAergic synaptic transmission (by ~50%), which suggests how the putative hyperactivity of this area, as described above, might arise from a reduced inhibition rather than an intrinsic membrane origin or an increase in glutamatergic drive. Additional analysis using evoked inhibitory currents highlights how the reduced inhibitory drive may be explained by a reduced release probability in inhibitory synapses onto pyramidal neurons in KO neurons. This reduced inhibition could be explained by a reduced release probability in inhibitory inputs onto KO neurons [73].

Due to methodological restrictions, we were bound to using a mixed cohort of male and female mice, which could dilute the effect, since there are sex differences in neuronal excitability of mPFC [74]. Furthermore, younger mice were used, which required using a milder stress protocol. In future studies, same sex- and age-matched subjects should be used for assessing electrophysiological properties of the mPFC of Arc-NAPE-PLD KO mice.

In summary, our study provides compelling evidence for the involvement of AEA signaling in modulating anxiety-like phenotypes and indicates that targeting of the eCB system provides a very promising and translationally relevant approach for developing successful therapies in the context of stress-induced psychiatric disorders. In particular for the prevention of the emergence of such disorders, when treatment starts immediately after the stressful event.

## Supplementary information

Text Suppl Figure

Suppl Figure 1

Suppl Figure 2

Suppl Figure 3

Suppl Figure 4

Suppl Figure 5

Suppl Figure

## Data Availability

The raw data presented in this study are available on request from the corresponding author.

## Code availability

Analysis code is available on request from the corresponding author.
