# Supplementary material for: NAPE-PLD deletion in stress-TRAPed neurons results in an anxiogenic phenotype
Source: Transl Psychiatry. 2023 May 6;13:152. doi: 10.1038/s41398-023-02448-9 (PMC10164145; doi:10.1038/s41398-023-02448-9)
Supplement: Supplementary file 1 — Text Suppl Figure [file 41398_2023_2448_MOESM1_ESM.docx]

**Supplementary Figure 1.** **Behavioral assessment of stressed Arc-NAPE-PLD WT and KO mice. A-C:** Group comparison of Arc-NAPE-PLD WT and KO in standard behavioral tests. SI: social interaction test **(A)**, nesting **(B)**, and TST: tail suspension test **(C)**. Unpaired t test was used to identify significant differences. **D:** Bodyweight of Arc-NAPE-PLD WT and KO mice during 14 days of CSD paradigm and on the first day of behavioral testing (day 21). ANOVA with multiple comparisons was used to identify significant differences. Data represented as mean ±SEM. WT/KO n=16/24.

**Supplementary Figure 2. Behavioral assessment of non-stressed Arc-NAPE-PLD WT and KO mice. A-D:** Group comparison of Arc-NAPE-PLD WT and KO in standard behavior tests. SI: social interaction test **(A)**, LDT: light-dark test **(B)**, EPM: elevated plus maze **(C)**, and nesting **(D)**. Unpaired t test was used to identify significant differences. Data represented as mean ±SEM. WT/KO n=16/24.

**Supplementary Figure 3. PEA (A) and 2-AG (B) levels in brain regions of stressed Arc-NAPE-PLD WT and KO mice measured by LC/MS.** Genotype and brain region differences were assessed using linear mixed effects models. Genotype and brain region were included as fixed effects in the model and animal as random effect. Pair-wise comparisons were based on estimated model means with Tukey p value adjustment in case of multiple comparisons. Asterisks indicate significant differences between genotype in a specific region: * p˂0.05, *** p˂0.001. Letters indicate the significance of pairwise comparisons of different brain regions within the same genotype. Groups sharing the same letter are significantly different from each other at p˂0.05. Data are represented as mean ±SEM and individual values; WT/KO n=16/24. Abbreviations: PFC, prefrontal cortex; dHip, dorsal hippocampus; vHip, ventral hippocampus; Hypo, hypothalamus, LC/MS, liquid chromatography/mass spectrometry.

**Supplementary Figure 4. Correlation matrix between behavioral outcome measures and molecular markers in four brain regions.** Correlations were calculated using the Pearson correlation coefficient. Positive correlations appear as blue dots, whereas negative correlations are colored red. The diameter of the dot and the color intensity indicate the strength of the correlation. Non-significant correlations (p>0.05) are not shown in the matrix.

**Supplementary Figure 5. Confirmation of successful Arc-mediated recombination induced in the modified social defeat stress procedure.** The genomic PCR was performed on the brain slices used for electrophysiological experiments from Arc-NAPE-PLD KO and WT mice injected with TAM and subjected to modified social defeat stress. DNA isolation was performed using a standard method with proteinase K tissue lysis and subsequent isopropanol/ethanol DNA extraction. **A:** Primers used for PCR and expected band sizes. **B:** PCR showing Arc-Cre allele (tg) at 250 bp. **C:** PCR for detection of NAPE-PLD floxed allele. Band at 299 bp indicates Arc-Cre mediated deletion in the NAPE-PLD floxed allele (recombined allele). **B-C:** Lanes 1 and 4 contain DNA with genotype Arc-Cre^tg/wt^; NAPE-PLD^fl/fl^; lanes 2 and 3 contain DNA with genotype Arc-Cre^wt/wt^; NAPE-PLD^fl/fl^ mouse. Marker: 766, 500, 300, 150, 50 bp; bp – base pair.

**Supplementary Figure 6.** **Passive and active membrane properties. A-D:** Statistical analysis of passive membrane properties in neurons from WT and KO mice. Resting membrane potential **(A)**, membrane capacitance **(B)** and membrane time constant **(C)** were not significantly different between WT and KO, while membrane resistance **(B)** was significantly increased in neurons from KO mice as compared to WT. Statistical significance was evaluated using Mann-Whitney U test, * p < 0.05. **E-I:** Statistical evaluation of active membrane properties in WT and KO neurons. Amplitude of action potentials was significantly higher in KO than in WT cells **(H)**, while threshold **(G)**, duration **(I)** and latency of the first action potential **(E, F)** were not different between the genotypes. Statistical significance was evaluated using Student´s t test, * p < 0.05. No differences could be found in firing frequency after application of current steps of increasing amplitude.
