# Supplementary figures and images for: NAPE-PLD deletion in stress-TRAPed neurons results in an anxiogenic phenotype
Source: Transl Psychiatry. 2023 May 6;13:152. doi: 10.1038/s41398-023-02448-9 (PMC10164145; doi:10.1038/s41398-023-02448-9)

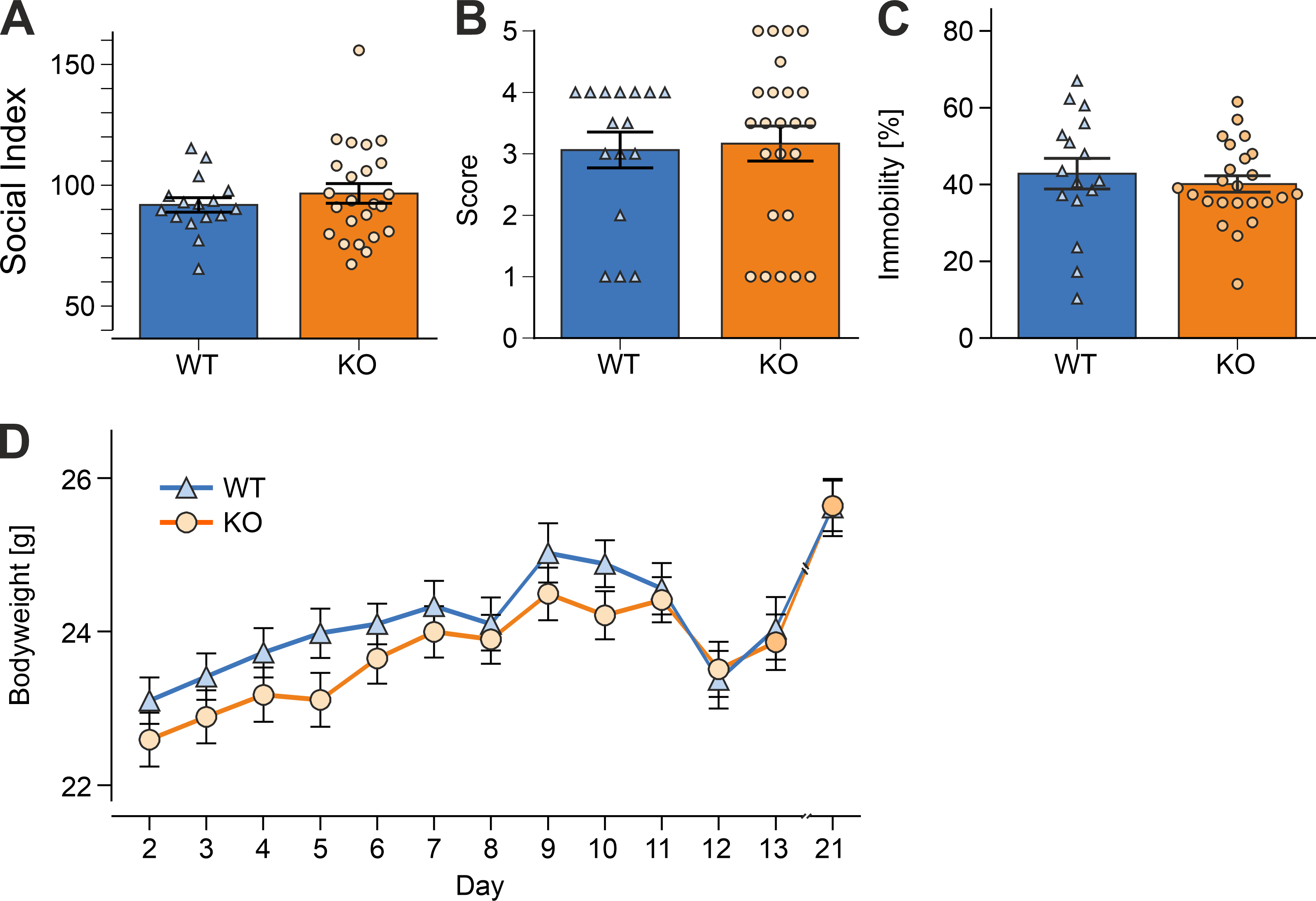

Supplement: Supplementary file 2 — Suppl Figure 1 [file 41398_2023_2448_MOESM2_ESM.tif]

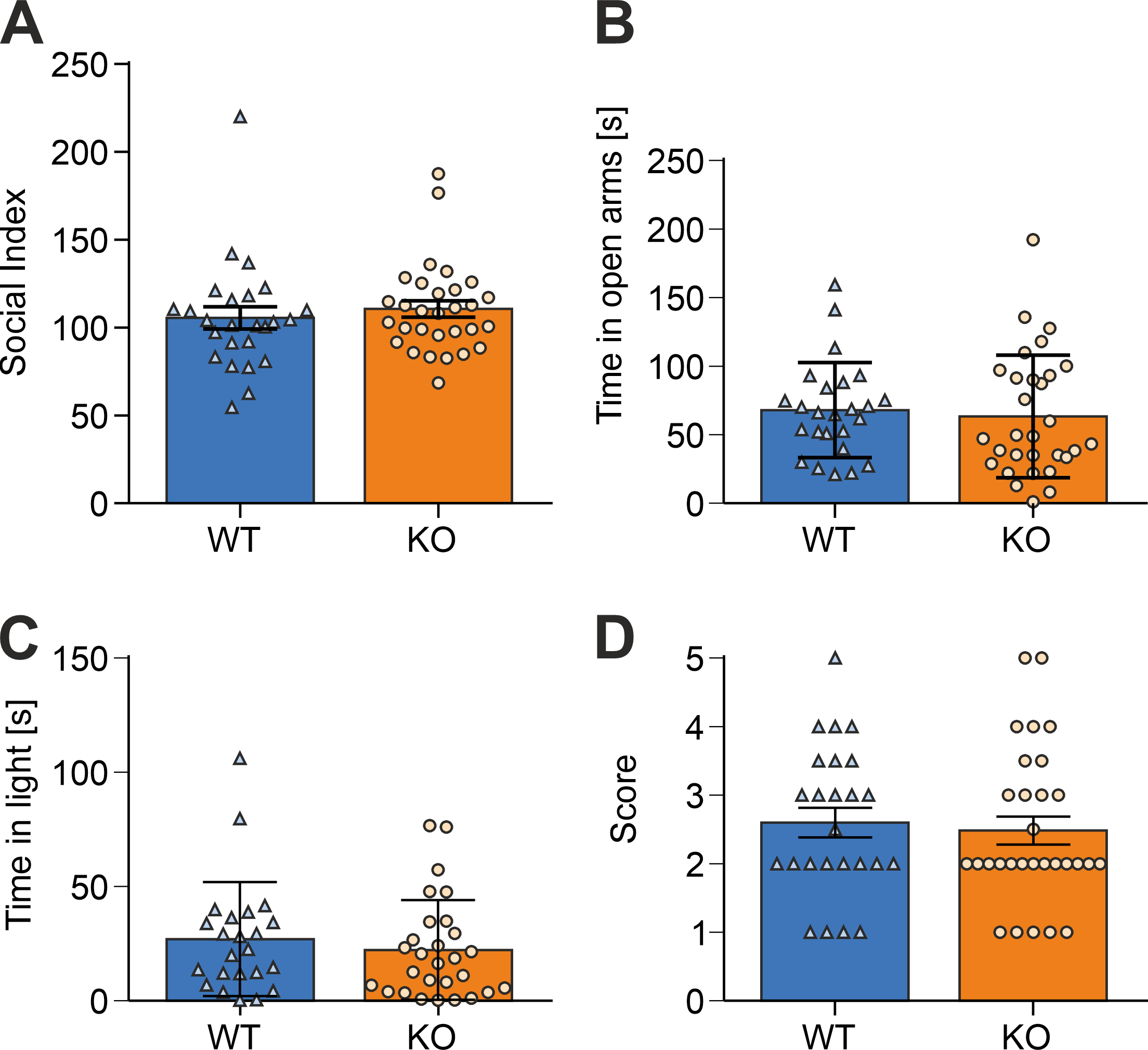

Supplement: Supplementary file 3 — Suppl Figure 2 [file 41398_2023_2448_MOESM3_ESM.tif]

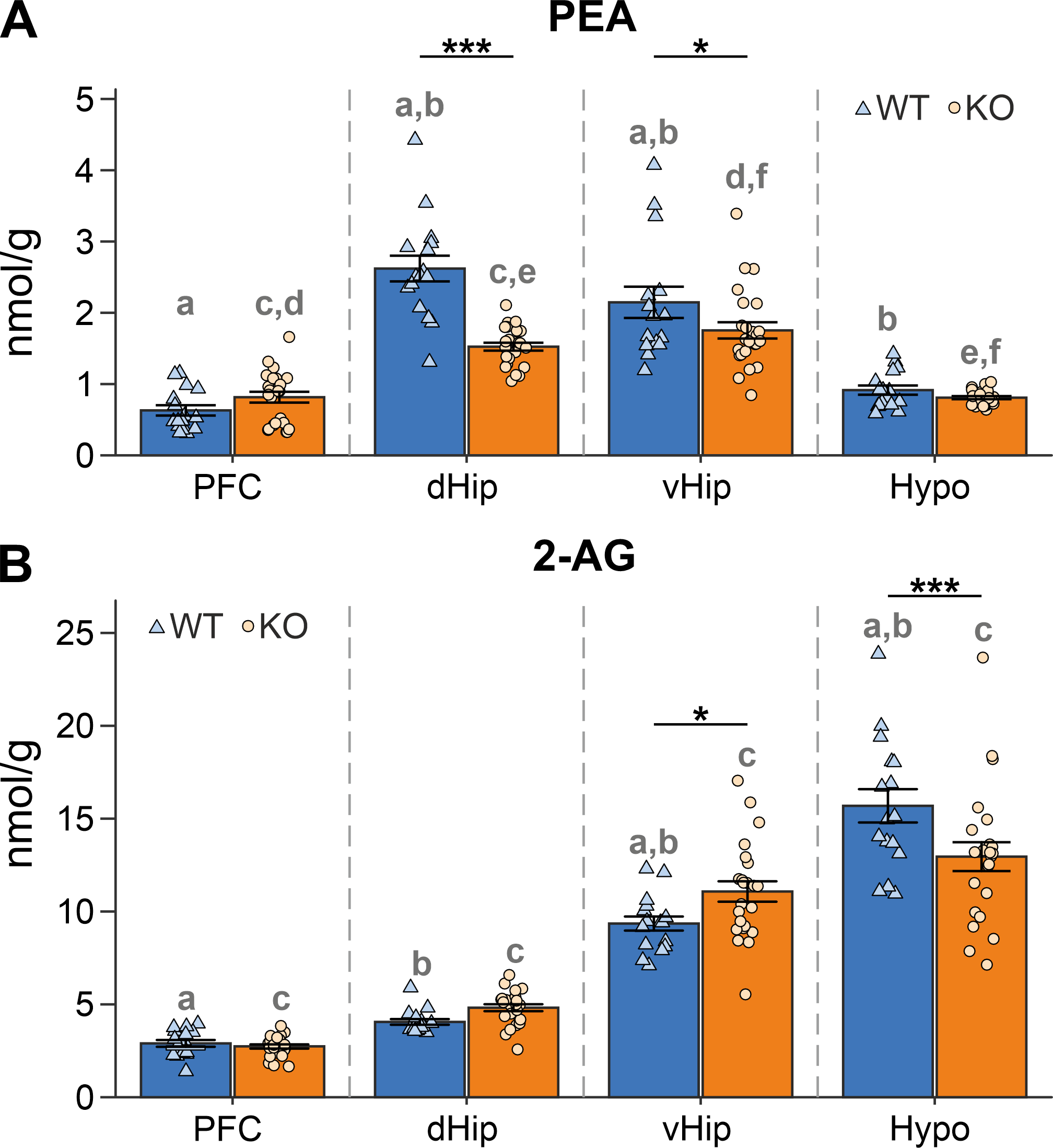

Supplement: Supplementary file 4 — Suppl Figure 3 [file 41398_2023_2448_MOESM4_ESM.tif]

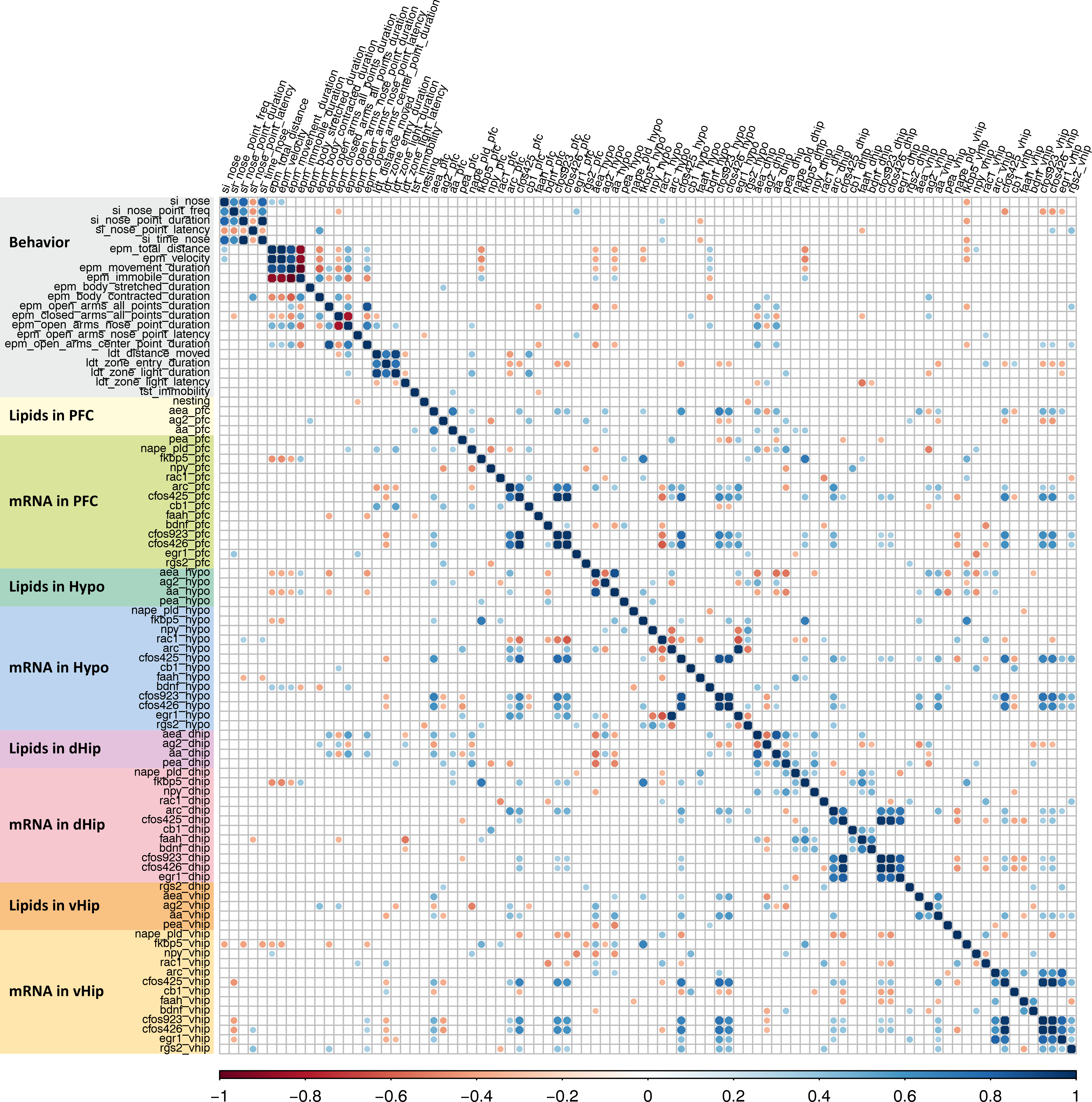

Supplement: Supplementary file 5 — Suppl Figure 4 [file 41398_2023_2448_MOESM5_ESM.tif]

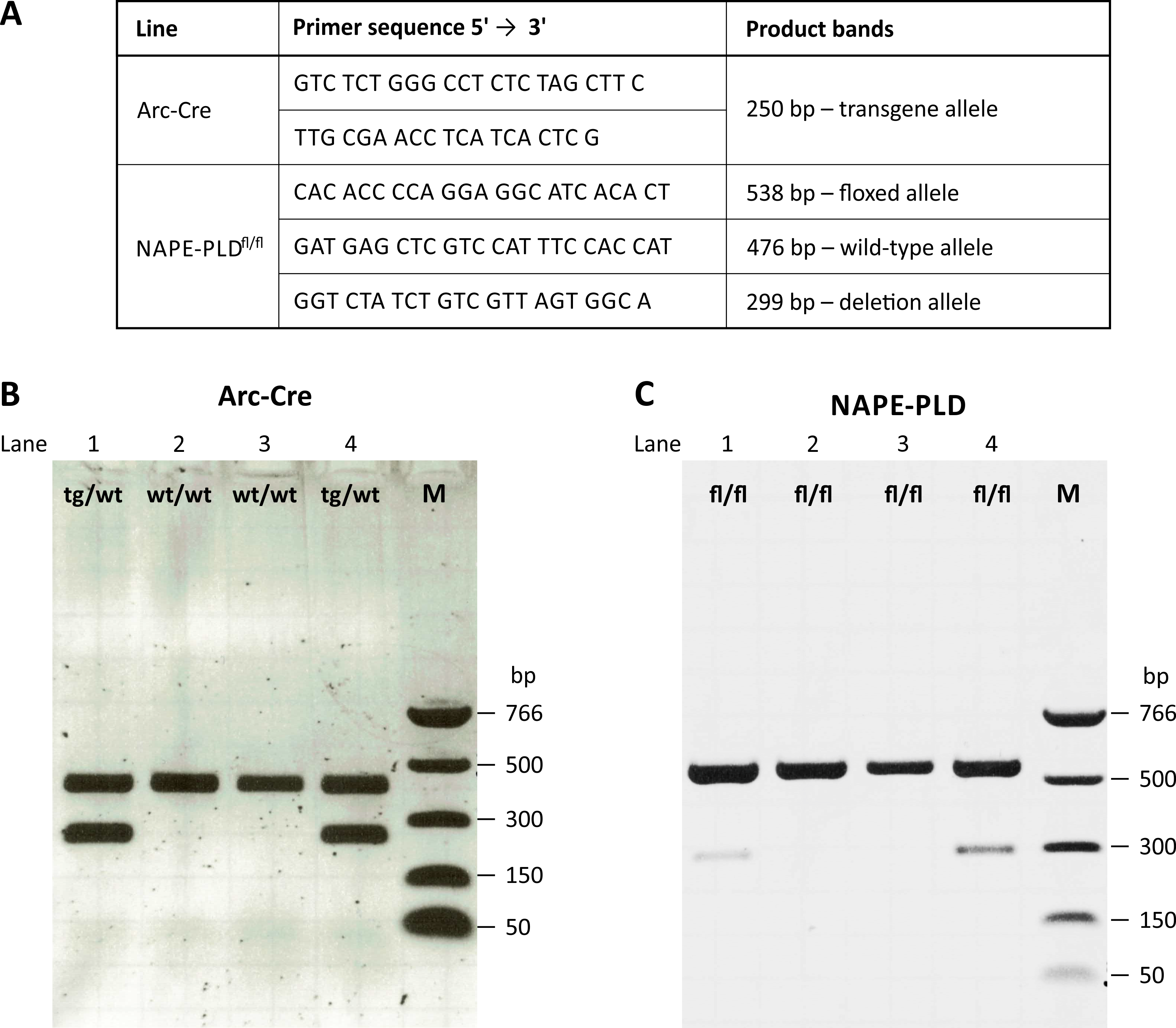

Supplement: Supplementary file 6 — Suppl Figure 5 [file 41398_2023_2448_MOESM6_ESM.tif]

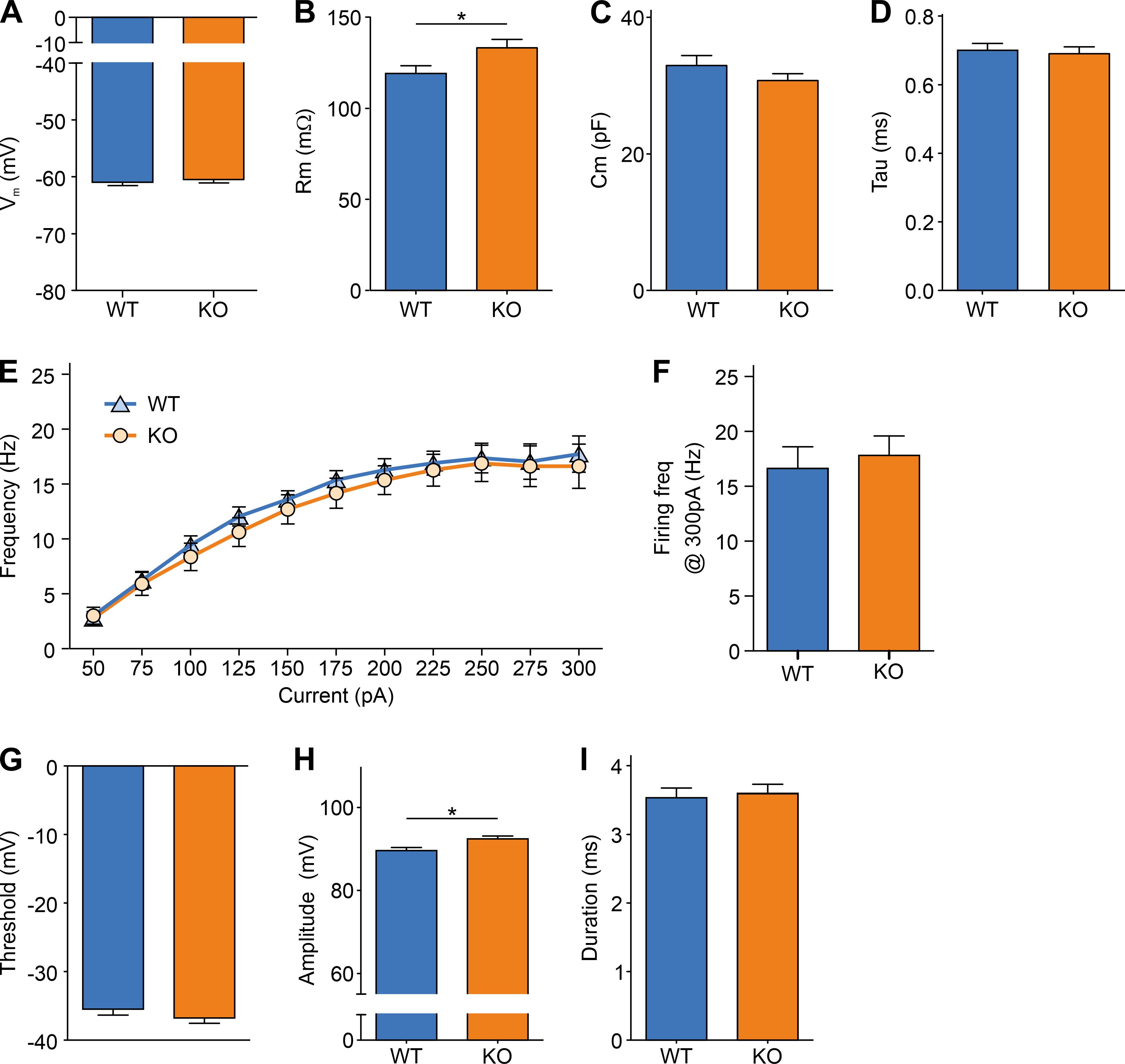

Supplement: Supplementary file 7 — Suppl Figure [file 41398_2023_2448_MOESM7_ESM.tif]
